# Perivascular tumor-associated macrophages and their role in cancer progression

**DOI:** 10.1042/EBC20220242

**Published:** 2023-09-28

**Authors:** Meriem Bahri, Joanne E. Anstee, James W. Opzoomer, James N. Arnold

**Affiliations:** School of Cancer and Pharmaceutical Sciences, King’s College London, Faculty of Life Sciences and Medicine, Guy’s Hospital, London SE1 1UL, United Kingdom

**Keywords:** cancer, macrophages, metastasis, perivascular, polarization, tumor microenvironments

## Abstract

Perivascular (Pv) tumor-associated macrophages (TAMs) are a highly specialized stromal subset within the tumor microenvironment (TME) that are defined by their spatial proximity, within one cell thickness, to blood vasculature. PvTAMs have been demonstrated to support a variety of pro-tumoral functions including angiogenesis, metastasis, and modulating the immune and stromal landscape. Furthermore, PvTAMs can also limit the response of anti-cancer and anti-angiogenic therapies and support tumor recurrence post-treatment. However, their role may not exclusively be pro-tumoral as PvTAMs can also have immune-stimulatory capabilities. PvTAMs are derived from a monocyte progenitor that develop and localize to the Pv niche as part of a multistep process which relies on a series of signals from tumor, endothelial and Pv mesenchymal cell populations. These cellular communications and signals create a highly specialized TAM subset that can also form CCR5-dependent multicellular ‘nest’ structures in the Pv niche. This review considers our current understanding of the role of PvTAMs, their markers for identification, development, and function in cancer. The role of PvTAMs in supporting disease progression and modulating the outcome from anti-cancer therapies highlight these cells as a therapeutic target. However, their resistance to pan-TAM targeting therapies, such as those targeting the colony stimulating factor-1 (CSF1)-CSF1 receptor axis, prompts the need for more targeted therapeutic approaches to be considered for this subset. This review highlights potential therapeutic strategies to target and modulate PvTAM development and function in the TME.

## Introduction

**Figure 1:**
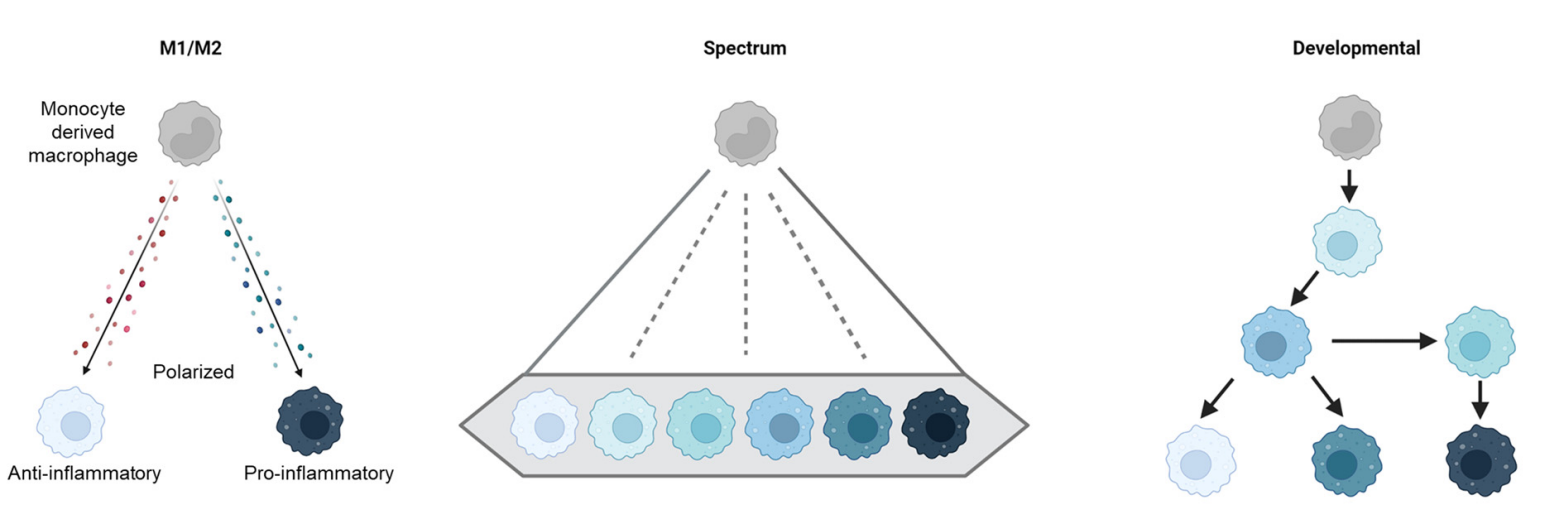
Models for TAM polarization The dichotomy of the conventionally defined ‘M1’ and ‘M2’ macrophage polarization program, as pro- (M1) and anti- (M2) inflammatory stages of their phenotypic program, has served as a valuable framework for understanding and classifying the extremes of TAM phenotypes (left). However, a more inclusive ‘spectrum’ polarization model was proposed to enable the capturing of inter- and intra-tumoral TAM phenotypes identified (middle). Recent advances in our knowledge of TAM development, have highlighted that the most specialized TAM phenotypes develop in a multistep process and, as such, we propose a third ‘developmental’ model which encompasses both the heterogeneity of TAM phenotypes and the potential linkages of these phenotypes into developmental pathways within the TME (right). Although these models are most easily applied to monocyte-derived macrophages, it should be noted that self-renewing tissue-resident macrophages can also get incorporated into a growing tumor and contribute to TAM heterogeneity which are not reflected in the above schematics.

Macrophages are a phenotypically and functionally heterogeneous population of cells within the tumor microenvironment (TME) [1–3]. Heterogeneity within the tumor associated macrophage (TAM) population can be derived from their cellular origin [4], environmental cues [3,5,6] and their spatial location within the TME [7,8]. The dichotomy of the classically described ‘M1’ and ‘M2’ macrophage polarization programs [9], as pro- (M1) and anti- (M2) inflammatory states of their phenotypic program (Figure 1), have provided a useful template for understanding and categorizing the extremes of TAM functionality. However, the inflexibility of this model for capturing all inter- and intra-tumoral TAM phenotypes described, in particular where a TAM displays both M1 and M2-associated markers resulted in a more inclusive ‘spectrum’ polarization model being proposed [10] (Figure 1). There is growing evidence that TAM development is potentially a multistep process [6,11,12], suggesting that phenotypic diversity might also arise from intermediary stages of TAM development within the TME and, as such, we propose that a developmental view to TAM specialization might be appropriate (Figure 1). In the spontaneous mouse mammary tumor virus (MMTV)-polyoma middle T antigen (PyMT) driven murine model of breast cancer, pseudo-temporal trajectory analyses of single cell RNA sequencing (scRNA-seq) data from the total TAM population predicted that TAM diversity could be refined into a tri-directional TAM developmental program within the TME, resulting in three highly polarized/specialized TAM extremes [11]. As such, TAM phenotypic identity in the tumor is not a binary response but could be most accurately modeled by a developmental process guided by the TME. Understanding these developmental paths, their signals and transcription factors is vital to tackling how to therapeutically target and manipulate these cells *in vivo*, particularly as pro- and anti-tumoral TAM phenotypes co-exist in the TME [11,13].

One distinct subset of TAMs in the TME reside in the perivascular (Pv) niche [14]. PvTAMs are defined as macrophages which reside within 15–20 µm, or one cell thickness, from a blood vessel [3,11,14]. The accumulation of macrophages near vasculature is not cancer or inflammation-specific and Pv macrophages have also been observed in healthy tissues where they have been described to play a range of homeostatic functions associated with the vasculature [14,15]. In cancer, PvTAMs have emerged as a therapeutic target due to their pro-tumoral functions associated with neo-angiogenesis [11,16], metastasis [3,6], the immunological ‘heat’ of the tumor [12,13], mesenchymal cell expansion [11], and limiting the response of anti-cancer therapies [12,17], which facilitate disease progression. However, there is also recent evidence that PvTAMs can play an immune-stimulatory role in the TME [13], highlighting the need to understand this TAM subset in greater detail to define how to therapeutically target/modulate the population in an optimal manner. Anti-colony stimulating factor 1 receptor (CSF1R) blocking antibodies act as a therapeutic strategy to deplete TAMs from the TME, but have shown limited therapeutic clinical efficacy as a monotherapy strategy [18]. Interestingly, PvTAMs have been demonstrated to be resistant to therapies targeting this axis [19,20], highlighting the need to consider more specific therapeutic approaches for targeting this population. This review will discuss our current understanding of the role of PvTAMs, their markers, development, and functions in cancer progression.

## PvTAM markers and heterogeneity

Several protein markers have been used to distinguish PvTAMs, however, the spatial proximity of the TAM to the vasculature remains the primary distinguishing feature of these cells. In the *MMTV-PyMT* murine model of breast cancer, PvTAMs preferentially express CD206 (Macrophage Mannose Receptor) [7,8,11], a c-type lectin scavenger receptor [21,22]. CD206^high^ PvTAMs express low levels of Arginase-1 (Arg-1) [8], a classical ‘M2’ TAM marker. However, as the spatial position of the TAM distances away from the ‘well nourished’ Pv microenvironment (where there is reducing oxygen tension and higher lactate levels) the TAMs display a switched CD206^low^Arg-1^high^ phenotype, which is also associated with high expression of vascular endothelial growth factor (VEGFA) [8]. Although, it should be noted that VEGFA is also expressed by PvTAMs [6].

Expression of the angiopoietin receptor, TIE2, has been widely utilized to distinguish a pro-angiogenic and pro-metastatic PvTAM population [23,24]. TIE2-expressing macrophages co-express VEGFA, CD206, CD11b, and F4/80 but are negative for CD11c in the Pv niche [23]. TIE2 also marks a progenitor subset of monocytes in the peripheral blood that develop into TIE2^+^ PvTAMs [25]. The protein TIE2 plays a functional role in the recruitment of the TAM subset to the Pv niche through its interaction with its ligand, angiopoietin-2, which acts as a chemotactic signal [25]. As such, TIE2 is a therapeutic target of the PvTAM subset where pharmacological blockade of TIE2 signaling using rebastinib can reduce the abundance of TIE2^+^ PvTAM within the tumor [26].

Expression of the hyaluronan receptor, lymphatic vessel endothelial hyaluronan receptor-1 (LYVE-1), marks a population of monocyte-derived Pv macrophages that can be found across several tissue types in both humans and mice [27–29]. LYVE-1 expressing PvTAMs have been described in the *MMTV-PyMT* mouse model where they can be labeled by their phagocytic uptake of extravascular dextran [30,31] or fluorescently-labeled liposomes [11]. These TAMs express CD206 and high levels of the anti-apoptotic and immune suppressive enzyme heme oxygenase-1 (HO-1) [11]. HO-1 is an enzyme which breaks down heme into the biologically active catabolites biliverdin, ferrous iron, and carbon monoxide (CO) [32,33] and several studies have highlighted its association with PvTAMs [3,12,17]. Folate receptor 2 (FOLR2/FOLR β) has recently been demonstrated to mark a PvTAM population that co-expresses LYVE-1, CD206 and MHCII [13]. FOLR2^+^ PvTAMs bear a high similarity to tissue resident Pv macrophages and may either be directly derived from these cells in the TME or could be subject to niche-dependent transcriptional imprinting [13,27]. How FOLR2^+^ and LYVE-1^+^ populations relate to the TIE2^+^ PvTAMs have yet to be established; however, *Tie2*/*Tek* gene expression was not detectable in bulk RNAseq data from LYVE-1^+^ TAMs in *MMTV-PyMT* tumors [12], suggesting they are discrete populations.

**Figure 2:**
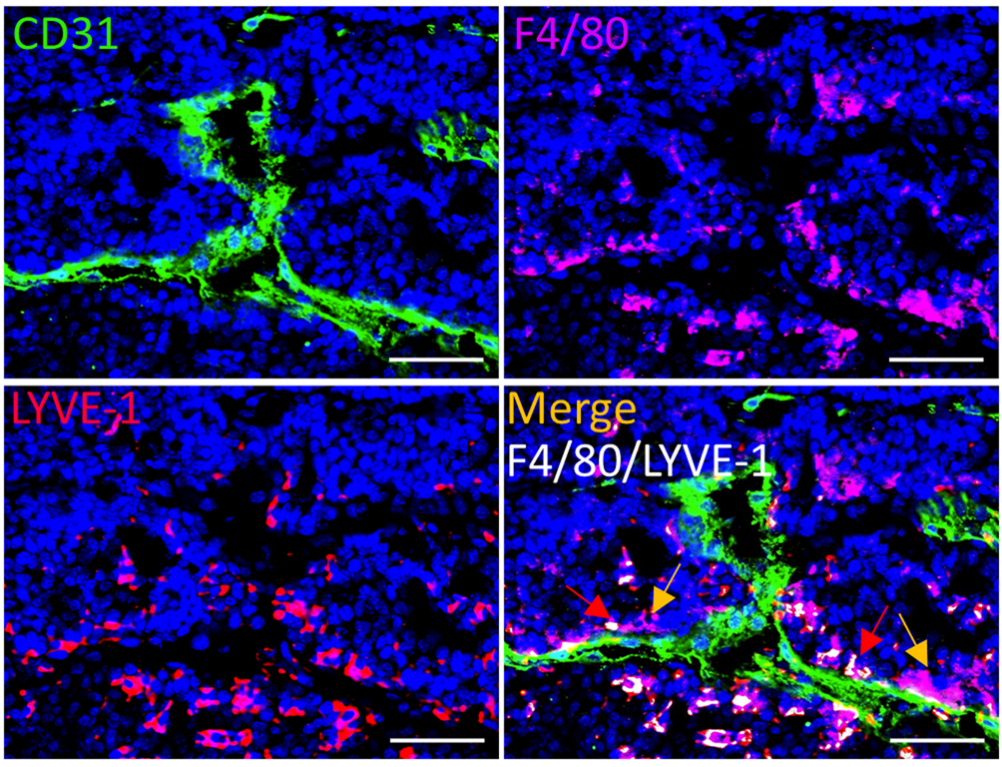
Evidence of TAM heterogeneity and nest formation in the Pv niche Representative image of a frozen section of tumor from the spontaneous *MMTV-PyMT* murine model of breast cancer [66], stained with DAPI (nuclei;blue) and antibodies against F4/80 (magenta), LYVE-1 (red) and CD31 (green). Colocalizing pixels for F4/80 and LYVE-1 is shown in white. Red arrows highlight examples of LYVE-1^+^ PvTAMs and yellow arrows highlight examples of LYVE-1^−^ PvTAMs within the TME. Scale bar: 25 µm.

Several markers have been used to identify PvTAMs, however the heterogeneity of the population needs to be fully resolved. As both LYVE-1^+^ and LYVE-1^−^ TAMs can be found in the PV niche (Figure 2), it suggests that heterogeneity within the population exists, which might reflect either distinct subsets of these cells in the niche or a less mature PvTAM phenotype. With the dawn of spatial ‘omic’ based approaches and multiplexed imaging technologies [34], the tools are now available to dissect the full diversity of PvTAM phenotypes.

## PvTAM development

**Figure 3:**
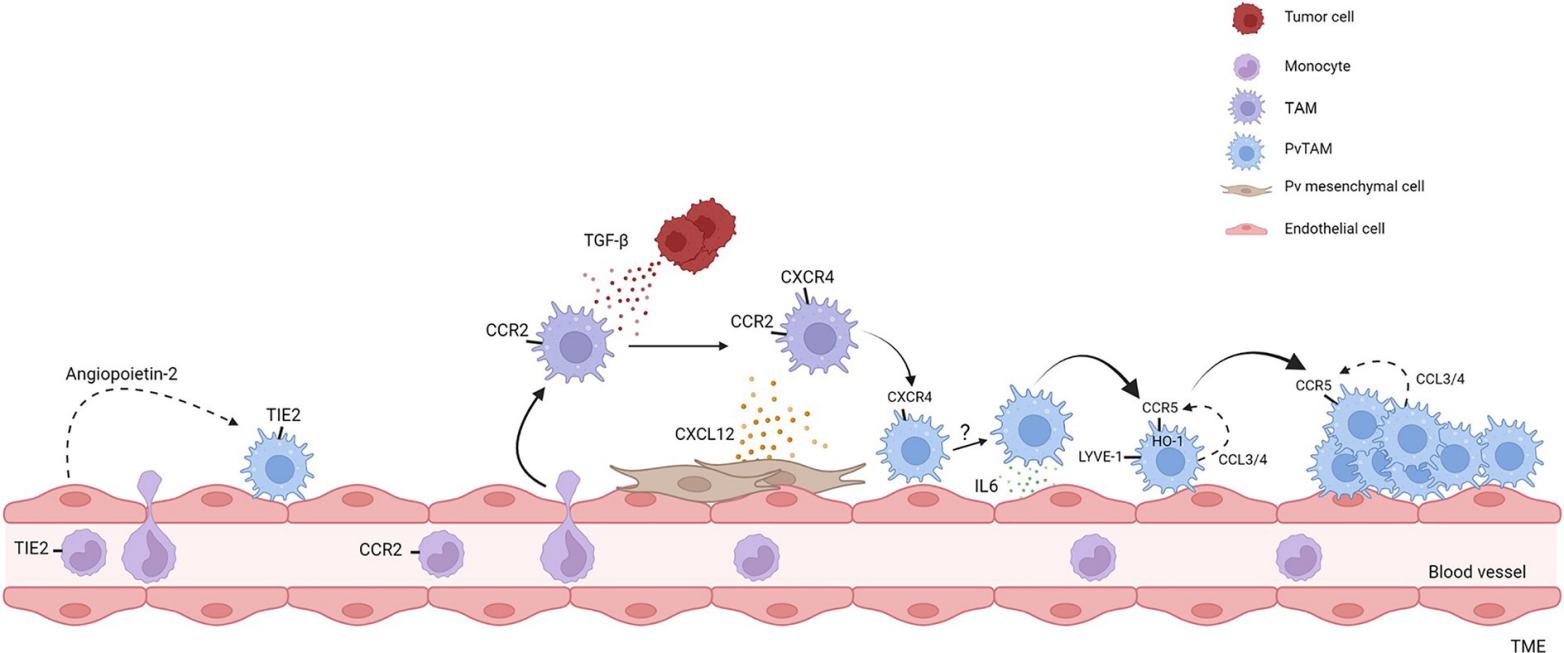
Development of PvTAMs Summary of the developmental pathways of PvTAMs highlighted in the manuscript text. TIE2^+^ PvTAMs can arise from TIE2 expressing monocytes recruited from the peripheral blood. TIE2^+^ PvTAMs are retained in the Pv niche through their interaction with angiopoietin-2 expressed by endothelial cells. Additionally, CCR2-expressing monocytes enter the tumor and develop into PvTAMs. Upon entering the tumor, TAMs respond to TGF-β derived from tumor cells which up-regulate CXCR4. CXCR4^+^ TAMs then migrate towards CXCL12 expressed by Pv mesenchymal cells to reach the Pv niche. IL-6 expressed by the endothelium polarizes PvTAMs to develop a LYVE-1^+^CCR5^+^HO-1^+^ phenotype. LYVE-1^+^ PvTAMs express CCL3 and CCL4 allowing the subset to communicate via CCR5 to form multicellular nests in the Pv niche. Black arrows denote differentiation and dashed arrows represent ligand/receptor interactions. It is currently unknown if LYVE-1^+^CCR5^+^HO-1^+^ PvTAM, CXCR4^+^, and TIE2^+^ PvTAM populations are related or if they are discrete phenotypic and functional populations.

PvTAMs are a specialized and highly polarized TAM phenotype [11], and several studies have linked PvTAMs to a peripheral monocytic origin [6,12,24,27]. As such, PvTAMs develop and polarize within the TME. Recent studies have highlighted PvTAM development to not be a binary one-step process but a developmental program guided by the TME through intermediate phenotypes from their blood-derived CCR2^+^ monocyte progenitor (Figure 3) [6,11,12], supporting the broader need to consider TAM heterogeneity within a ‘developmental’ model context (Figure 1). Arwert et al*.* elegantly demonstrated that post-conditional depletion of the TAM population (using the MaFIA (Macrophage Fas-Induced Apoptosis) mouse model [35,36]), repopulation of PvTAMs took 10–14 days to return to baseline levels. By contrast, the total TAM abundance in the tumor reached baseline 4–5 days post-depletion [6]. CCR2^+^ monocytes/macrophages were demonstrated to adopt a ‘migratory’ phenotype and localize to the Pv niche through chemotaxis via CXCR4 towards a CXCL12 gradient [6] (Figure 3). However, the TIE2 receptor may also play a role in this process directing migration towards angiopoietin-2 expressed by the endothelium [25]. CXCR4 has been identified as a key chemokine receptor in locating TAMs to the Pv niche. Pharmacological inhibition of CXCR4 using AMD3100 prevents PvTAM accumulation at the vasculature [6,17] and as such, provides a therapeutic opportunity to target their development. TAM expression of CXCR4 is induced by tumor cell-derived transforming growth factor-β (TGF-β) which enables the migration of these TAMs to the endothelium which is orchestrated by Pv fibroblasts, phenotypically distinct from pericytes (desmin^−^), which express high levels of CXCL12 [6]. Within the Pv space LYVE-1^+^ PvTAMs form a tight niche with pericyte-like (PDGFRα^low^PDGFRβ^+^NG2^+^desmin^+^αSMA^+^) mesenchymal cells by secreting platelet derived growth factor-C (PDGF-C) [11] (Figure 3), highlighting a potential synergistic communication between TAMs and mesenchymal cells in the development and maintenance of the mature Pv niche. As mesenchymal cells have been demonstrated to be plastic in their phenotype [37,38], whether PvTAMs influence a pericyte population or elicit a pericyte-like transcriptional program from the desmin^−^ CXCL12^+^ mesenchymal cells upon reaching the Pv niche remains an interesting question to address.

Recently we demonstrated LYVE-1^+^ PvTAMs are reliant on IL-6 to guide their phenotypic identify in a STAT3/c-MAF dependent signaling pathway [12]. Endothelial cells are the primary source of IL-6 within the niche, which acts to direct the maturation of the transcriptional program of these cells by up-regulating LYVE-1, CD206, and HO-1 expression [12]. Also, this IL-6 maturation signal drives expression of the chemokine receptor CCR5 which connects a communication axis between LYVE-1^+^ PvTAMs (which co-express the CCR5 ligands CCL3 and CCL4) enabling their ability to form multicellular nests in the Pv niche (Figure 3). These ‘nests’ and their expression of HO-1 have been demonstrated to be associated with the functionality of these cells in the resistance to chemotherapy and immune exclusion [12]. These data highlight the complexity of the formation of PvTAMs in the TME and their reliance on both the tumor cells and stromal cells to guide their developmental program.

## Role of PvTAMs in angiogenesis

**Figure 4:**
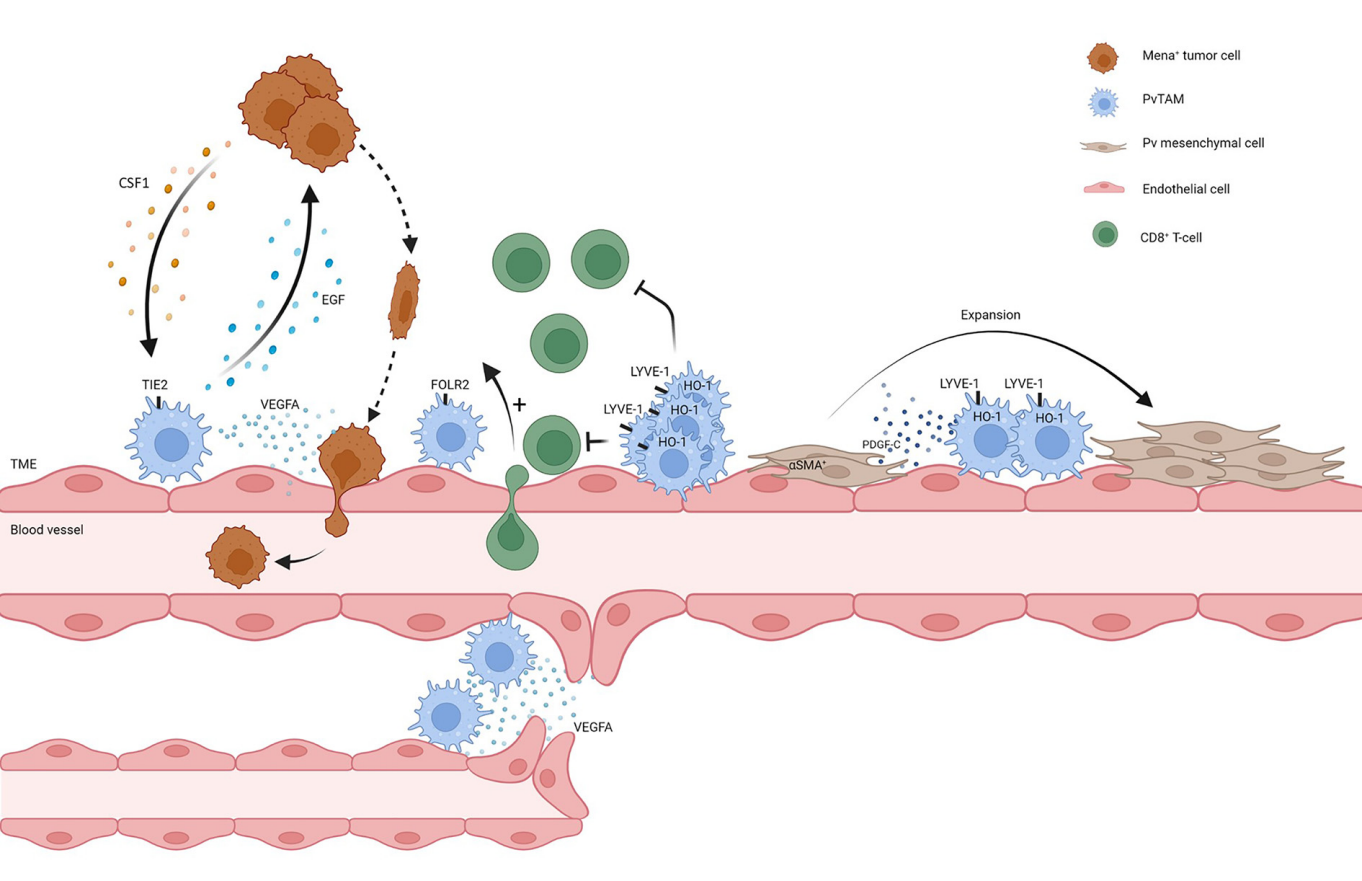
PvTAM functions in the TME Summary of the functions of PvTAMs highlighted in the manuscript text. PvTAMs can facilitate intravasation of Mena^+^ tumor cells into the circulation, where PvTAMs express EGF and tumor cells express CSF1 and participate in a cross-communication axis, which results in streaming of tumor cells to the Pv niche. PvTAMs facilitate intravasation of tumor cells through their secretion of VEGFA which results in temporary vessel leakiness. Additionally, PvTAMs are associated with an emerging role in modulating the immune landscape of the TME, where there are reports that populations of PvTAMs are associated with exclusion (Lyve1^+^HO1^+^ PvTAMs) and recruitment (FOLR2^+^ PvTAMs) of CD8^+^ T-cells into the tumor. LYVE-1^+^ PvTAMs orchestrate a selective expansion of a pericyte-like mesenchymal cell population through their expression of PDGF-C, creating a pro-angiogenic niche. PvTAMs, can facilitate neo-angiogenesis and anastomosis of blood vessels in the TME.

Neo-angiogenesis is vital to tumor progression and PvTAMs have been demonstrated to shape the process through engaging in communication axes with endothelial cells and mesenchymal populations in the Pv niche [39]. In particular, TIE2-expressing PvTAMs have been well characterized for their pro-angiogenic functions [24]. The abundance of this TIE2^+^ PvTAM has been correlated with microvascular density and metastasis in several types of human cancer [23,25]. In a developmental context, TIE2^+^ PvTAMs facilitate blood vessel anastomosis (the joining of two blood vessels) through their secretion of VEGFA [40] (Figure 4). TIE2^high^CD206^+^CXCR4^high^ macrophages have also been demonstrated, in a variety of murine models of cancer, to preferentially accumulate at the vasculature post-chemotherapy treatment and facilitate tumor-relapse through their role in promoting re-vascularization of the tissue orchestrated by their release of VEGFA [17]. This is not a chemotherapy-specific response and TIE2-expressing PvTAMs also accumulate at the tumor vasculature post treatment of vasculature disruptive agents such as combretastatin A4 phosphate [41] and anti-VEGF therapies [42] as well as post irradiation [43] which limits the efficacy of these therapies. Pharmacological inhibition of CXCR4 using AMD3100 to prevent PvTAM accumulation reduced tumor recurrence in a murine Lewis Lung carcinoma model post cyclophosphamide treatment [17], providing a therapeutic strategy to block these effects. Highlighting the need to consider combination therapies to deplete or modulate PvTAMs to improve the efficacy of anti-cancer therapies. Anti-colony stimulating factor 1 receptor (CSF1R) blocking antibodies act as a therapeutic strategy to deplete TAMs from the TME [18,20]. However, pro-angiogenic VEGFA^+^ PvTAMs have been demonstrated to be largely resistant to this therapy approach in a mouse model of colorectal cancer (CRC) [19]. This emphasizes the need to consider therapies such as AMD3100 to target PvTAM accumulation thereby indirectly blocking their pro-angiogenic capabilities.

We recently demonstrated that LYVE-1^+^ PvTAMs orchestrate the formation of a pro-angiogenic niche with a population of pericyte-like mesenchymal cells displaying both cancer associated fibroblast (CAF) and pericyte markers (PDGFRα^low^ PDGFRβ^+^NG2^+^desmin^+^αSMA^+^) [11]. Pericytes are a population of specialized vessel-associated mesenchymal cells which support the angiogenic process through contributing to vessel stabilization and endothelial cell survival [16,44,45]. Interestingly, LYVE-1^+^ PvTAMs orchestrated the selective expansion of the pericyte-like population within the niche through their expression of the growth factor PDGF-C which signaled through PDGFRα expressed by the pericyte-like population [11] (Figure 4). An elegant study by Shook et al*.* demonstrated that macrophage expression of PDGF-C was also implicated in supporting the expansion of αSMA^+^ myofibroblast populations in the wound healing response [46], a stromal reaction which parallels that of cancer as a ‘wound that does not heal’ [3,47]. These data collectively highlight a fundamental role for PvTAMs in facilitating angiogenesis in cancer which can limit the efficacy of anti-cancer and anti-angiogenic therapies and promote disease recurrence through their pro-angiogenic functions, emphasizing the need to therapeutically target PvTAMs to achieve optimal therapeutic responses.

## PvTAMs and the immunological ‘heat’ of the tumor

There is an emerging role for PvTAMs in modulating the immunological ‘heat’ of the tumor; however, their role is not entirely resolved. PvTAM populations have both been associated with immune exclusion [12] and immune-stimulatory functions [13] of T-cells in the TME. In an experimental bacterial meningitis model, PvTAM depletion revealed these cells to be critical in allowing leukocyte influx across the blood–brain barrier, which had a key protective role in this setting [48]. We recently demonstrated that a population of LYVE-1^+^MHCII^lo^CD206^hi^ HO-1^high^ PvTAM in *MMTV-PyMT* tumors form CCR5-dependent multicellular nests within the Pv niche which are associated with immune exclusion of CD8^+^ T-cells from the TME through a mechanism dependent on their expression of the HO-1 enzyme [12] (Figure 4). HO-1 activity in the TME also influences the immune-modulatory effects of chemotherapy that are dependent on CD8^+^ T-cells [12,49]. In contrast, a recent study by Nalio Ramos et al. demonstrated that in human breast cancer that a FOLR2^+^CADM1^−^HLA-DR^+^ PvTAM subset (which co-express *LYVE-1* and *MRC1*) was associated with CD8^+^ T-cell infiltration into the TME and identified a role for these cells in priming CD8^+^ T-cell effector function [13]. Colocalization of FOLR2^+^ TAMs/CD8^+^ T-cells correlates with favorable clinical outcomes, suggesting an anti-tumorigenic role for the FOLR2^+^ TAM subset [13] (Figure 4). The PvTAMs characterized by Nalio et al*.* expressed MHCII, whereas LYVE-1^+^ PvTAMs associated with an immune exclusion role expressed low MHCII, highlighting a subset or context specific functionality of these cells, potentially differentiated based on their MHCII expression. As such, the role of PvTAMs in relation to the anti-tumor immune response requires further investigation and may reveal therapeutic opportunities for modulating the immune landscape of the TME.

## Role of PvTAMs in metastasis

Metastasis is a complex multistep process which accounts for greater than 90% of cancer-related mortalities. The role of PvTAMs in cancer metastasis has been widely established, and these cells both facilitate the intravasation event at the primary tumor [2,23] and also the extravasation and seeding of disseminated tumor cells at secondary sites [50–52]. One of the most well-documented roles of PvTAMs is facilitating intravasation of malignant tumor cells into the circulation [23,53] (Figure 4). Metastasis promoting PvTAMs reside in what has been termed ‘Tumor MicroEnvironments of Metastasis’ (TMEM), which involves a PvTAM, tumor cell expressing a splice variant of mammalian-enabled protein ‘Mena’ referred to as Mena invasive (Mena^INV^), and an endothelial cell which are in direct contact. Mena is an epidermal growth factor (EGF)-responsive cell migration protein which is a member of the Ena/VASP family of actin-binding proteins and is a mediator of cytoskeletal rearrangement, which enhances tumor cell morphology and motility [54]. Mena is expressed in tumor cells which successfully invade into the circulation [54]. The importance of Mena is well established, and Mena-null *MMTV-PyMT* mice have longer tumor latencies, less circulating tumor cells and fewer metastasis compared with Mena-wild-type *MMTV-PyMT* mice [55]. TMEMs are specialized niches which represent sites through which Mena-expressing tumor cells intravasate into the blood stream [53,56].

Macrophages and tumor cells participate in a cross-communication paracrine loop in which TAMs express EGF which signals on tumor cells, while reciprocally tumor cells express CSF1 which promotes macrophage chemotaxis, differentiation, and survival [2,57–59]. This reciprocal interaction facilitates co-ordinated ‘streaming’ of tumor cells toward blood vessels [60], delivering tumor cells to TMEMs where PvTAMs facilitate the intravasation event [53,57,61] (Figure 4). PvTAMs facilitate intravasation through their secretion of VEGFA which creates a localized, and transient, loss of vascular junctions and temporary vessel leakiness [23]. TMEMs have been observed in human tumors and their density predicts distant metastatic recurrence in patients with breast cancer [62–64]. Furthermore, in the facilitation of metastasis, TIE2-expressing PvTAMs have been demonstrated to release matrix metalloproteinase 9 (MMP9), which can promote the invasion of tumor cells [42] and TIE2 can facilitate the transendothelial migration event [26]. How TMEMs form, and if they are the same niche as the Pv multicellular nests of LYVE-1^+^ PyTAMs, remain to be determined.

## Conclusions

PvTAMs have emerged as an important subset of TAMs within the TME orchestrating a range of pro-tumoral functions [65], highlighting the need to investigate the modulation and targeting of these cells in the therapeutic setting. The protracted multistep nature of their development from monocytic progenitors to mature PvTAMs [6], and their collaboration to form multicellular nests [12], provides opportunities for therapeutic intervention and selective targeting of this population. A deeper understanding of the molecular basis behind why PvTAM populations are more resistant to clinically used therapeutics targeting the CSF1/CSF1R axis remains an important question to address. The full heterogeneity of the PvTAM population requires further investigation to establish inter- and intra-tumoral phenotypic and functional differences of these cells which will help to potentially rationalize their pro- and anti-tumoral roles in the TME. The interaction of PvTAMs and mesenchymal populations in the Pv niche [6,11] highlights the need to further consider the orchestrating roles of these cells in the stromal response in cancer and their broader role in shaping the stromal reaction. As such, therapeutically targeting PvTAMs may also provide collateral benefits in unwinding the broader stromal response in cancer. The unique spatial location of this highly specialized TAM subset at the vasculature endows these cells with a ‘gatekeeper’ role within the TME and their relative resistance to current pan-TAM targeted therapies further emphasizes the need for utilizing novel targeted strategies to therapeutically target PvTAMs and their role in cancer progression.

## Summary

- PvTAMs influence neo-angiogenesis, metastasis and the stromal response in cancer which can influence the outcome of anti-cancer therapies and contribute to disease progression.
- Several markers have been associated with PvTAMs such as TIE2, CD206, LYVE-1, HO-1, and FOLR2; however, their full inter- and intra- tumoral heterogeneity remain to be fully determined.
- PvTAMs are derived from a monocyte progenitor and develop through a multistage developmental process in the TME, which can also result in their formation into CCR5-dependent multicellular nests.
- PvTAMs represent a therapeutic target in cancer but how to target the population most effectively in the clinic remains to be determined.

## Open Access

Open access for this article was enabled by the participation of King's College London in an all-inclusive *Read & Publish* agreement with Portland Press and the Biochemical Society under a transformative agreement with JISC.

## Abbreviations

CADM1: cell adhesion molecule 1
CAF: cancer-associated fibroblast
CSF1: colony stimulating factor 1
CSF1R: colony stimulating factor 1 receptor
EGF: epidermal growth factor
FOLR2: folate receptor 2
HO-1: heme oxygenase 1
IL: interleukin
LYVE-1: lymphatic vessel endothelial hyaluronan receptor-1
MaFia: macrophage fas-induced apoptosis
MMP9: matrix metalloproteinase 9
MMTV: mouse mammary tumor virus promoter
NO: nitric oxide
PDGF: platelet-derived growth factor
PDGFR: platelet-derived growth factor receptor
Pv: perivascular
PvTAM(s): perivascular tumor-associated macrophage(s)
PyMT: polyoma middle T-antigen
scRNA-seq: single-cell RNA sequencing
TAM(s): tumor associated macrophage(s)
TGF-β: transforming growth factor-β
TLR: toll-like receptor
TME: tumor microenvironment
TMEM: tumor microenvironment of metastasis
TNFα: tumor necrosis factor-α
VEGFA: vascular endothelial growth factor A
